# p62 aggregates mediated Caspase 8 activation is responsible for progression of ovarian cancer

**DOI:** 10.1111/jcmm.14288

**Published:** 2019-04-02

**Authors:** Xiao‐Yu Yan, Xin‐Ru Zhong, Si‐Hang Yu, Li‐Chao Zhang, Ya‐Nan Liu, Yong Zhang, Lian‐Kun Sun, Jing Su

**Affiliations:** ^1^ Department of Pathophysiology, College of Basic Medical Sciences Jilin University Changchun Jilin P.R. China

**Keywords:** apoptosis, autophagy, Caspase 8, ovarian cancer, p62

## Abstract

Increasing evidence suggests that p62/SQSTM1 functions as a signalling centre in cancer. However, the role of p62 in tumour development depends on the interacting factors it recruits and its precise regulatory mechanism remains unclear. In this study, we investigated the pro‐death signalling recruitment of p62 with the goal of improving anti‐tumour drug effects in ovarian cancer treatment. We found that p62 with Caspase 8 high expression is correlated with longer survival time compared with cases of low Caspase 8 expression in ovarian cancer. In vivo experiments suggested that insoluble p62 and ubiquitinated protein accumulation induced by autophagy impairment promoted the activation of Caspase 8 and increased cell sensitivity to cisplatin. Furthermore, p62 functional domain UBA and LIR mutants regulated autophagic flux and attenuated Caspase 8 activation, which indicates that autophagic degradation is involved in p62‐mediated activation of Caspase 8 in ovarian cancer cells. Collectively, our study demonstrates that p62 promotes Caspase 8 activation through autophagy flux blockage with cisplatin treatment. We have provided evidence that autophagy induction followed by its blockade increases cell sensitivity to chemotherapy which is dependent on p62‐Caspase 8 mediated apoptosis signalling. p62 exhibits pro‐death functions through its interaction with Caspase 8. p62 and Caspase 8 may become novel prognostic biomarkers and oncotargets for ovarian cancer treatment.

## 1 INTRODUCTION

p62/SQSTM1 is a receptor for ubiquitinated proteins that are degraded by autophagy. p62 serves as a multi‐functional hub that regulates molecules in the key signalling pathways in cancer.^1^ Recently, high p62 cytoplasmic expression was shown to be associated with poor prognosis; conversely, other investigators have indicated that p62 is positively correlated with cell death. For example, proteasome inhibitor MG132 was reported to promote apoptosis of U87 cells through p62 accumulation and metastatic and recurrent tumour tissues express low levels of p62.^2^, ^3^, ^4^ Our previous study found that p62 was involved in regulating the SKOV3 ovarian cancer cells sensitivity to chemotherapy through NF‐κB signalling and ubiquitin clearance.^5^, ^6^ Together these results indicate p62 has multi‐faced functions and plays complex roles in determining the outcome of tumourigenesis.

p62 is also a substrate of selective autophagy and its degradation is mainly regulated by autophagy.^7^ It is known that autophagosomes undergo maturation by fusion with lysosomes for degradation after targeted substrates are sequestered in the autophagosome. During this process p62 binds ubiquitinated proteins through its ubiquitin‐associated (UBA) domain and anchors to LC3 in the autophagosome membrane through its LC3‐interacting region (LIR) domain.^8^, ^9^ Recent evidence suggests that the completion of autophagy relies on smooth autophagic flux and renewal of autophagosomes whereas dysfunction in the autophagy pathway leads to the accumulation of p62 and damaged proteins that possibly results in cell death.^10^

Caspase 8 is a key protein in the extrinsic apoptotic pathway and its activation depends on oligomerization and self‐cleavage. Upon activation, full‐length Caspase 8 (p55/53) is cleaved into p43/41 and p10 fragments that are released into the cytoplasm to activate downstream caspases. The canonical Caspase 8 activation pathway is dependent on cell‐surface death receptors through the death‐inducing signalling complex (DISC)^11^ Thus, a recent study showed that TRAIL can induce p62‐dependent activation of Caspase 8 in H460 lung cancer cells.^12^ Furthermore, p62 accumulation promoted Caspase 8 activation in HCT116 cells treated with ABT263, an inhibitor of Bcl‐2.^13^ Therefore, exploration of the association between p62 and Caspase 8 might help elucidate the crosstalk between autophagy and apoptosis during ovarian cancer treatment.

Cisplatin is the first‐line agent used in the treatment of ovarian cancer. However, a high risk of tumour relapse and drug resistance are obstacles in its clinical use. Initial studies demonstrated that the anti‐tumour effects of cisplatin involved its interference with the DNA structure.^14^, ^15^ However, later reports showed that only approximately 1% of the cisplatin that enters into cells actually binds to DNA.^16^ The accumulated cisplatin in the cytoplasm may act as a stressor that affects the function of a variety proteins and signalling pathways including Caspase 8 and autophagy.^17^, ^18^

Here, we examined whether p62 exhibits pro‐death functions through its interaction with Caspase 8. Our data suggest high p62 and Caspase8 expression is correlated with longer survival time. Furthermore we provide evidence that p62 regulates autophagic flux through its autophagy‐related domains and suppression of autophagic flux promotes Caspase 8 recruitment and activation caused by p62 accumulation, which increases the sensitivity of ovarian cancer cells to cisplatin. Our results demonstrate that p62 and Caspase 8 may serve as potential prognostic biomarkers and oncotargets for individualized treatment of ovarian cancer patients.

## 2 MATERIALS AND METHODS

### 2.1 Cell lines and cell culture

SKOV3 and A2780 ovarian cancer cells were grown in RPMI‐1640 (Gibco Life Technologies, Carlsbad, CA) supplemented with 10% foetal bovine serum (Invitrogen, Carlsbad, CA) at 37°C at 5% CO_2 _concentration.

### 2.2 Reagents and antibodies

The reagents used in this study include the following: cisplatin (Sigma‐Aldrich, St. Louis, MO), 3‐(4,5‐dimethylthiazol‐2‐yl)‐2,5‐diphenyltetrazolium bromide (MTT) (Sigma‐Aldrich) and ViaFect™ transfection reagent (Promega, Madison, MI). Antibodies used in this study include anti‐p62 (Abcam, Cambridge, MA, USA), anti‐LC3 (Abcam), anti‐Caspase 8 (Proteintech, Chicago, IL), anti‐actin (Proteintech) and anti‐ubiquitin (Santa Cruz, CA).

### 2.3 Ovarian cancer tissues microarray and immunohistochemistry

A total of 160 ovarian cancer tissues were purchased from Shanghai Outdo Biotech Co.,Ltd., collected between 2009 and 2013 (Table 1). Immunohistochemistry of tissues was carried out using primary antibodies against p62 (Abcam) and Caspase 8 (Proteintech). Sections were fixed with 4% (v/v) formaldehyde in PBS and then dehydrated and embedded in paraffin. Tissues were sliced into 5 µm sections and antigen retrieval was performed by heating for 15 minutes in a microwave. Horse serum albumin (5%) was used to block non‐specific interaction. Sections were incubated with primary antibodies and then HRP‐conjugated secondary antibodies; DAPI was used to stain nuclei. Images were acquired using an Olympus microscope. For image analysis, Image Pro Plus 6.0 was used. The areas of total ovarian tumours and interested protein‐positive integrated option density (IOD) were quantified. After calculating the quotient of IOD and total area, the median value was used to divide patients into ‘high expression’ and ‘low expression’ groups.

**Table 1:** Clinical pathological characteristics of ovarian cancer cases

Characteristic	GC, *N* = 90	%
Age（y）		
< 65	140	87.5
≥ 65	20	12.5
miss	0	0
Tumour Size (cm)		
< 5	23	85.6
≥ 5	137	14.4
miss	0	0
Histological grade		
I‐III	57	35.6
III	71	44.4
miss	32	20
T stage		
T1‐T2	46	28.8
T3	112	70
miss	2	1.2
N stage		
N0	116	72.5
N1	42	26.3
miss	2	1.2
M stage		
M0	125	78.2
M1	33	20.6
miss	2	1.2
Relapse		
Absence	31	19.4
Presence	127	79.4
miss	2	1.2
Follow‐ups		
Dead	85	53.1
Survival	75	46.9
miss	0	0

### 2.4 Plasmids and transfection

The pcDNA3.1 vector (NC), pcDNA3.1‐p62, pcDNA3.1‐ΔUBA‐p62, pcDNA3.1‐L417V UBA‐p62 and pcDNA3.1‐W338A LIR‐p62 were purchased from Sangon Biotech (Shanghai, China). Cells were transfected using ViaFect™ transfection reagent according to the manufacturer's instructions.

### 2.5 Cytotoxicity assays

Cells (8 × 10^3^ cells per well) were plated in 96‐well plates and treated with drugs for 24 hours. MTT reagents were added and cells were incubated for 4 hours. Absorbance values were then measured at 570 nm using a Vmax Microplate Reader (Molecular Devices, LLC, Sunnyvale, CA).

### 2.6 Apoptosis assays

#### 2.6.1 Flow cytometry analysis

Cell death was analysed using Annexin‐V FITC/PI (BD Biosciences, Franklin Lakes, NJ) staining. Cells were seeded in 6‐well plates and treated with drugs as indicated. The attached and detached cells were harvested and subjected to Annexin‐V FITC/PI staining according to the manufacturer's instructions. Samples were analysed using an Accuri C6 Flow Cytometer (BD Biosciences).

#### 2.6.2 Caspase activity assay

Cells were plated in 96‐well plates. Caspase activity was evaluated using the Caspase‐Glo 8 Assay (Promega) according to the manufacturer's instructions. A microplate reader (FLUOstar Omega, BMG LABTECH, Germany) was used for detection.

#### 2.6.3 TUNEL assay

Cell apoptosis was determined using the TUNEL Apoptosis Detection Kit (Roche, Mannheim, Germany) following the manufacturer's protocol.

### 2.7 Cell fractionation

Cells were lysed in 1% Triton X‐100 PBS with protease inhibitors on ice for 30 minutes and then centrifuged for 30 minutes at 16,000 g. The insoluble fraction was lysed in 2% SDS with protease inhibitors at 60°C for 1 hour. Lysates were centrifuged for 30 minutes at 16,000 *g* and examined using immunoblotting.

### 2.8 Western blotting

Cells were lysed in RIPA buffer with protease inhibitors. Lysates were cleared by centrifugation at 1000 g for 15 minutes at 4°C, boiled in loading buffer and resolved using SDS‐PAGE. Proteins were transferred to PVDF membranes and membranes were blocked with 5% milk, followed by incubation with primary antibodies overnight at 4°C. Membranes were then incubated with HRP‐conjugated secondary antibodies (Proteintech). ECL reagent (Thermo Fisher Scientific, Rockford, IL) was used for immunodetection and visualization using Syngene Bio Imaging (Synoptics, Cambridge, UK).

### 2.9 Co‐immunoprecipitation

Cells were lysed in NP40 lysis buffer plus protease inhibitors. Lysates were incubated on ice for 30 minutes and cleared by centrifugation at 4500 rpm for 15 minutes at 4°C. Lysates were incubated with antibody overnight at 4°C, followed by incubation with 25 µL protein A and G agarose (Beyotime, China). Beads were washed three times with 1 ml PBS and bound complexes were analysed using immunoblotting.

### 2.10 Immunofluorescence and confocal microscopy

Cells were fixed in 4% (w/v) paraformaldehyde (PFA)/PBS for 20 minutes and then permeabilized with 0.1% Triton X‐100 for 15 minutes. After blocking with bovine serum albumin for 30 minutes, cells were incubated with primary antibody overnight at 4°C. Cells were then incubated with FITC/Texas Red‐conjugated secondary antibodies (Proteintech) at room temperature for 1 hour. The images were acquired using an Olympus FV1000 confocal laser microscope.

### 2.11 mCherry‐GFP‐LC3 for determining autophagic flux

SKOV3 cells stably expressing mCherry‐GFP‐LC3 were transfected with different p62 plasmids as indicated and then treated with cisplatin for 12 hours. Cells were fixed with 4% PFA, washed with PBS and analysed using fluorescence microscopy. Images were acquired randomly and analysed using Image J.

### 2.12 DQ Red BSA for determining lysosomal degradation

Cells were incubated with 20 µg/ml DQ Red BSA (DQ‐BSA; Life Technologies) for 30 minutes at 37°C followed by a 2 hour chase in full media with or without 25 µM chloroquine. Cells were then fixed with 4% PFA for 20 minutes at room temperature and analysed using fluorescence microscopy. Images were analysed by Image J.

### 2.13 GST pull‐down assay

Tumour tissues were lysed in RIPA buffer with protease and phosphatase inhibitors and then lysates were centrifuged at 4500 rpm at 4°C for 15 minutes. wt‐p62 and mutant p62 genes were inserted into the pGEX‐4T vector. The expression products were purified and coated with glutathione‐agarose beads. After incubating with lysates overnight at 4 °C, the beads were washed three times with PBS and suspended in loading buffer and boiled at 95°C for 5 minutes. The samples were analysed by immunoblotting with anti‐p62, anti‐Caspase 8 and anti‐LC3 antibodies.

### 2.14 In vivo xenograft experiment

All experimental procedures were performed in accordance with the National Institutes of Health guide for the care and use of laboratory animals. A2780 cells (5.0  ×  10^6^) were subcutaneously injected into the dorsal left or right flank of 4‐week‐old female BALB/C nude mice purchased from the Animal Experimental Center (Beijing, China). The animals were maintained under specific pathogen‐free conditions. Mice were randomized into four groups of three mice per group and the mice were intraperitoneally administered 3 mg/kg cisplatin and 35 mg/kg chloroquine every 2 days. The bodyweights and tumour volumes were recorded every day. Mice were sacrificed and tumours were dissected, weighed and photographed.

### 2.15 Immunohistochemistry

Tumour specimens from mouse xenografts were fixed in 4% (v/v) formaldehyde in PBS and then dehydrated and embedded in paraffin. Tissues were sliced into 5 µm sections and antigen retrieval was performed by heating for 15 minutes in a microwave. Horse serum albumin (5%) was used to block non‐specific interaction. Sections were incubated with primary antibodies and then HRP‐conjugated secondary antibodies; DAPI was used to stain nuclei. Images were acquired using an Olympus microscope.

### 2.16 Statistical analysis

Experimental data were presented as mean ± standard deviation (*SD*) and carried out using Student's *t *test. *P* < 0.05 was considered statistically significant. Statistical analysis was performed with GraphPad Prism 5 (La Jolla, CA).

## 3 RESULTS

### 3.1 The expression of p62 and Caspase 8 is correlated with prognosis of human ovarian cancer

**Figure 1:**
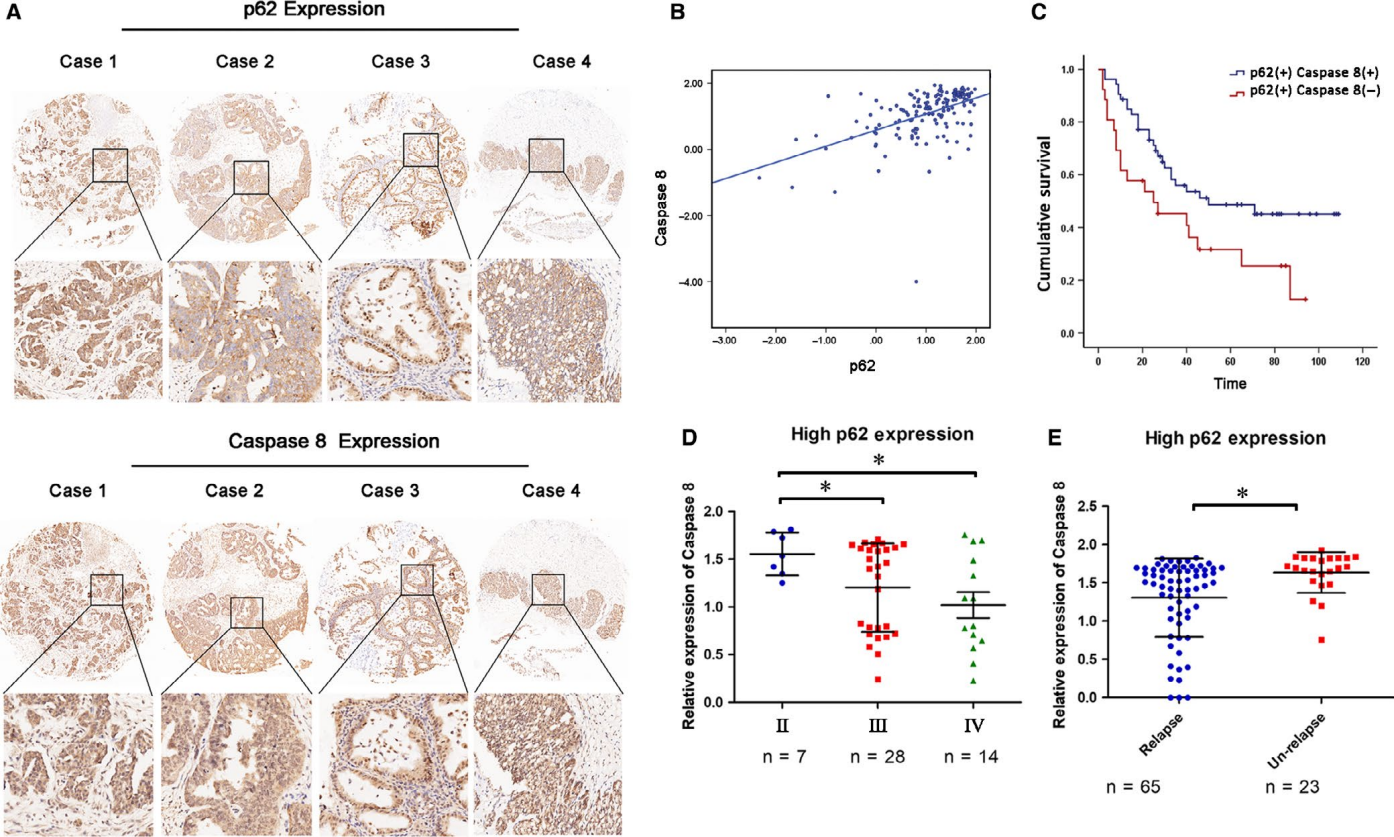
Expression of p62 and Caspase 8 in human ovarian cancer tissues. (A) Representative IHC for p62 and Caspase 8 expression in tissue microarrays (40×). Typical staining is shown in the adjacent rows (100×). (B) Spearman correlation analysis between p62 and Caspase 8 protein levels in human ovarian cancer tissues, ρ = 0.497, *P* < 0.01. (C) Kaplan‐Meier survival curve shows significant association between p62 and Caspase 8 expression and survival in ovarian cancer patients (*P* = 0.022). High p62 and high Caspase 8 (n = 53); High p62 and low Caspase 8 (n = 26). (D) Correlation between Caspase 8 expression and TNM stages in ovarian cancer tissues with p62 high expression. Statistical significance was determined using a two‐tailed, unpaired Student's *t* test.*P < 0.05. (E) The expression of Caspase 8 in p62‐overexpressing ovarian cancer tissues with and without relapse. Statistical significance was determined using a two‐tailed, unpaired Student's *t* test.*P < 0.05

Previous studies have examined p62 expression in ovarian cancer tissues.^2^, ^3^ We speculate that p62 exhibits pro‐survival or pro‐death functions depending on its interacting partners and thus we evaluated the expression of p62 and the apoptotic initiator Caspase 8 in human ovarian cancer tissues from 160 patients by immunohistochemical staining. Results showed 49.38% of the tumour tissues had increased p62 expression compared with the median value and Spearman correlation analysis confirmed that p62 expression was positively correlated with Caspase 8 (pho = 0.499, *P* < 0.01) (Figure 1A,B).

As we aimed to determine whether the recruitment of Caspase 8 could affect the role of p62 in ovarian cancer patients, we focused on ovarian cancer samples subtypes with high p62 expression/ high Caspase 8 expression and high p62/ low Caspase 8 expression. Kaplan‐Meier analysis suggested that patients with both p62 and Caspase 8 high expression had a significantly longer survival time than those with high p62 and low Caspase 8 expression (Figure 1C). Furthermore, we found that expression of Caspase 8 was negatively correlated with tumour‐node‐metastasis (TNM) stages in tissues with high p62 expression Figure 1D. In addition, high Caspase 8 expression was associated with less relapse risks in patients who overexpressed p62 (Figure 1E). Together, these results indicated that p62 and Caspase 8 may be prognostic factors for survival in ovarian cancer.

### 3.2 p62 accumulation and Caspase 8 activation induced by autophagy impairment increase the sensitivity of ovarian cancer cells to cisplatin

**Figure 2:**
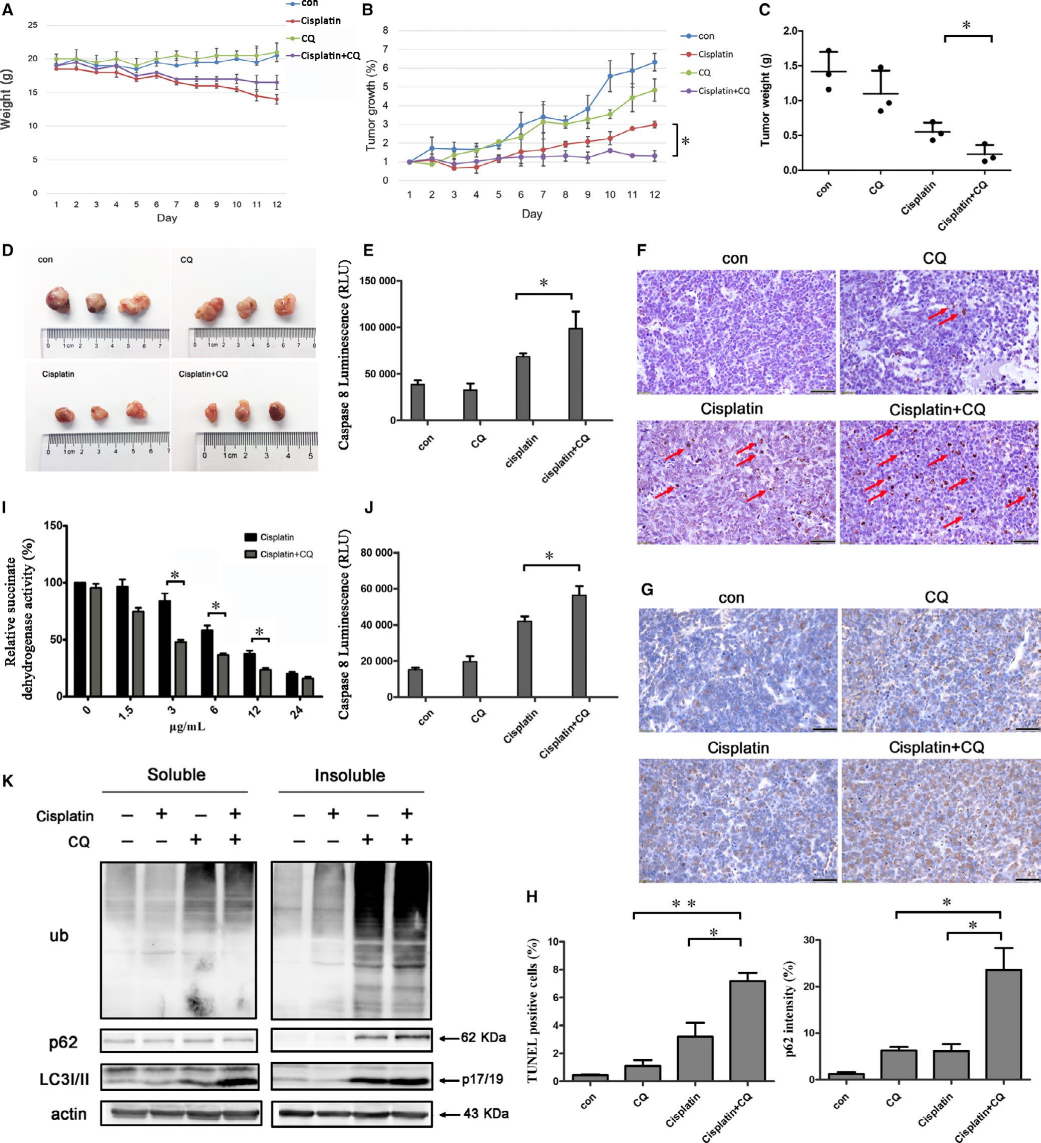
Chloroquine increased the sensitivity of ovarian cancer cells to cisplatin and accumulation of p62. (A‐C) Mouse xenograft model. A2780 ovarian cancer cells were subcutaneously implanted into nude mice. Mice were treated with 35 mg/kg chloroquine, 3 mg/kg cisplatin or the combination treatment for 12 days; control mice were treated with PBS (n = 3 per group). Bodyweight and tumour volume were measured daily; tumour volume was determined by measuring the length and width with calipers. On day 13, mice were killed and tumours were harvested. Statistical significance was determined using a two‐tailed, unpaired Student's *t* test.*P < 0.05. (D) Images of excised tumours from each treatment group. (E) Tumour tissues from mouse xenograft model were lysed in RIPAand the Caspase 8 activity was measured. Statistical significance was determined using a two‐tailed, unpaired Student's *t* test.*P < 0.05. (F) Representative images of TUNEL assay from mouse xenografts tumour specimens. Arrows indicate apoptotic cells. Scale bar, 50 µm. (G) Tumour specimens from mouse xenografts were subjected to immunohistochemical staining for p62 expression. Scale bar, 50 µm. (H) Left: Percentage of TUNEL positive cells in each sample in (F); Right: Quantification of p62 expression in (G), *P < 0.05, **P < 0.05. (I) SKOV3 ovarian cancer cells were treated with the indicated concentrations of cisplatin and chloroquine (25 µM) for 24 hours. Cell viability was measured using MTT assays. Data are presented as mean ± SD, n = 3. *P < 0.05. (J) SKOV3 cells were treated with cisplatin and CQ for 12 hours and the activity of Caspase 8 was analysed. Statistical significance was determined using a two‐tailed, unpaired Student's *t* test.*P < 0.05. (K) SKOV3 cells were treated with cisplatin (6 µg/ml) and/or chloroquine (25 µg/ml) for 12 hours. Cells were lysed in 1% Triton X‐100 and Triton soluble and insoluble fractions were immunoblotted for ubiquitin, p62, LC3 and β‐actin

To evaluate the effect of high expression of p62 and Caspase 8 in ovarian cancer, we established tumour xenografts by inoculating A2780 ovarian cancer cells in immune‐deficient BALB/C nude mice. After intraperitoneal administration with chloroquine, an autophagy inhibitor, cisplatin or both drugs for two weeks, we found that chloroquine improved the effect of cisplatin and inhibited tumour growth while activating Caspase 8 (Figure 2A‐E). TUNEL staining revealed that the combination of cisplatin and chloroquine significantly increased cell apoptosis compared with either treatment alone (Figure 2F,H). We confirmed that chloroquine promoted p62 accumulation (Figure 2G,H).

Consistent with the in vivo experiments, we found that chloroquine acts synergistically with cisplatin to induce cell death in SKOV3 cells in vitro (Figure 2I). A large number of p62 and ubiquitinated proteins accumulated as aggregates, which were observed in insoluble fractions (Figure 2K). The activity of Caspase 8 also increased (Figure 2J). These results suggested that p62 accumulation and Caspase 8 activation increased the sensitivity of ovarian cancer cells to chemotherapy.

### 3.3 p62 mediates Caspase 8 activation during cisplatin‐induced apoptosis in ovarian cancer cells

**Figure 3:**
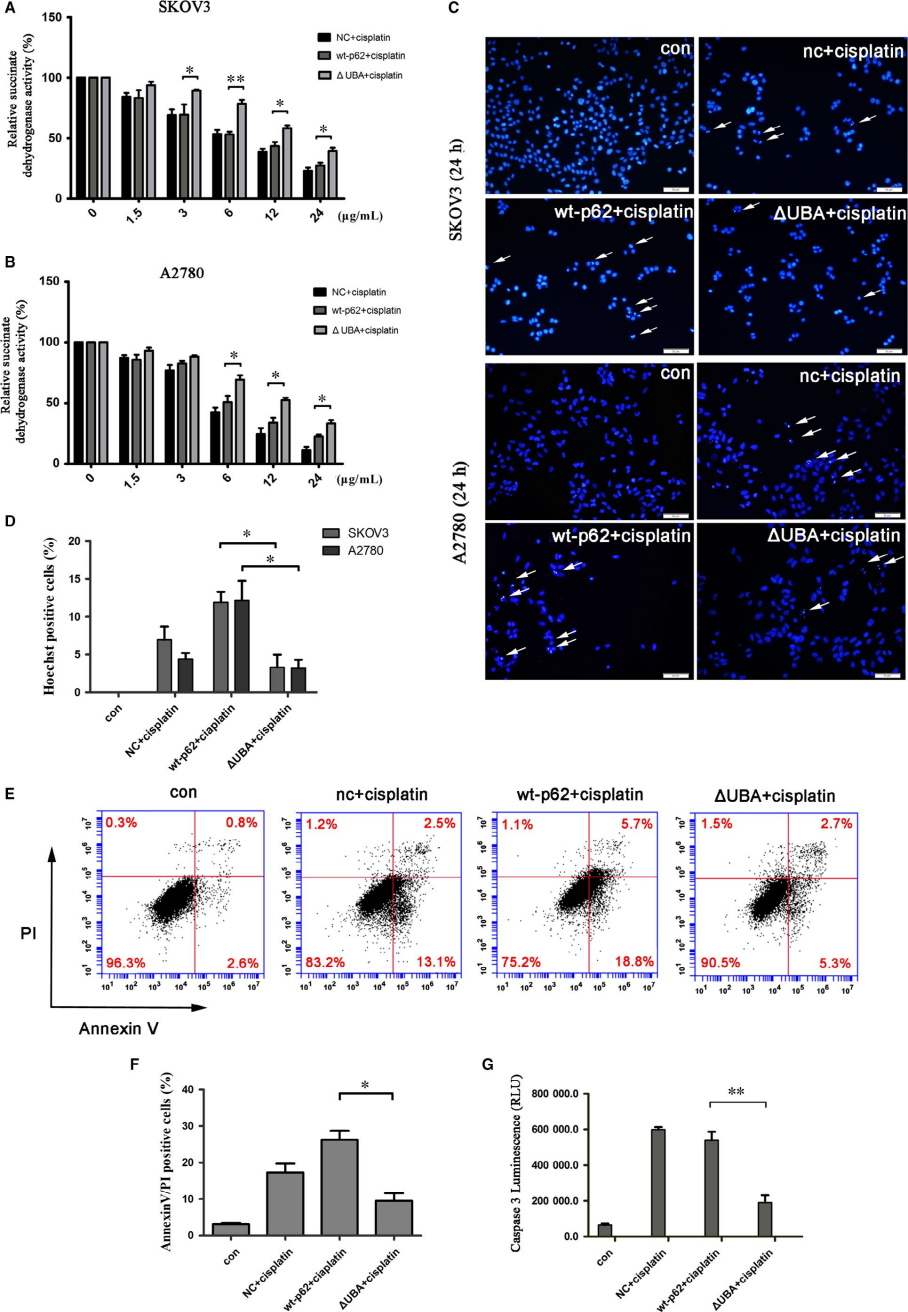
Abolishing the ability of p62 binding to ubiquitinated proteins decreases the sensitivity of ovarian cancer cells to cisplatin. (A,B), SKOV3 and A2780 cells were transfected with wt‐p62 or ΔUBA‐p62. After 24 hours, the cells were treated with varying doses of cisplatin. The viability of cells was analysed using MTT assay. Data are presented as mean ± SD, n = 3. *P < 0.05, **P < 0.01 vs wt‐p62. (C) SKOV3 and A2780 cells transfected with wt‐p62 or ΔUBA‐p62 were treated with 4.5 µg/ml or 6 µg/ml cisplatin for 24 hours and stained with Hoechst 33342. Cell morphology was observed using confocal microscopy. Arrows indicate apoptotic cells. Scale bar, 50 µm. (D) Percentage of Hoechst positive cells in each sample in (C), *P < 0.05. (E) A2780 cells were treated as in C and the apoptosis rate was detected by flow cytometry analysis.(F) Percentage of Apoptosis positive cells in each sample in (E), *P < 0.05. (G) A2780 cells were analysed using a Caspase3/7 activity assay kit. Data are presented as mean ± SD, n = 3. **P < 0.01 vs wt‐p62

**Figure 4:**
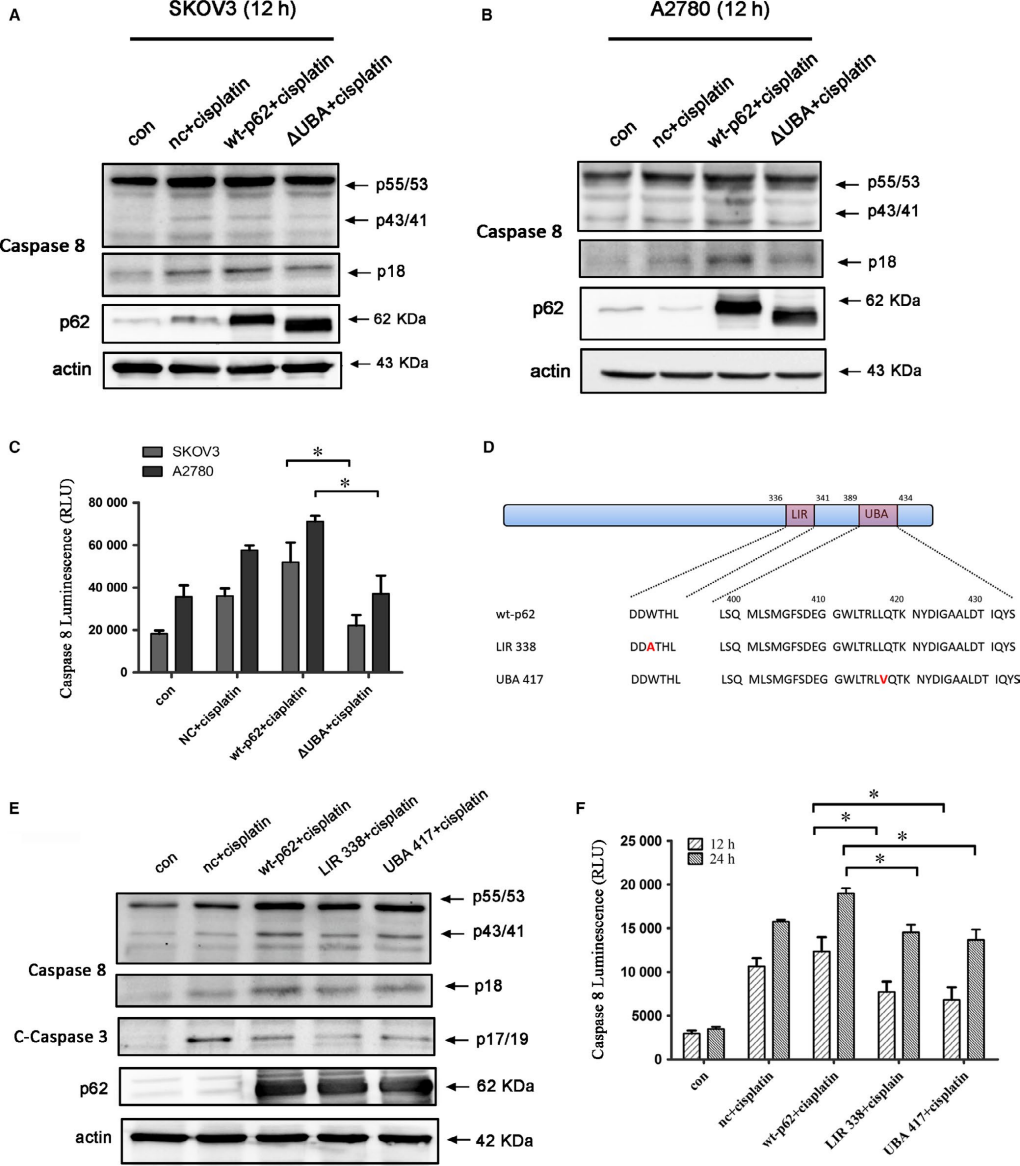
Cisplatin‐induced Caspase 8 activation is regulated by p62 in ovarian cancer cells. (A,B) SKOV3 and A2780 cells were transfected with wt‐p62 or ΔUBA‐p62. After 24 hours, cells were treated with 4.5 µg/ml or 6 µg/ml cisplatin for 12 hours and the expression of Caspase 8 was analysed by western blotting.(C) After the cells were treated as in A and B, the activity of Caspase 8 was analysed. Data are presented as mean ± SD, n = 3. **P < 0.01 vs wt‐p62. (D) Schematic representation of the p62 wild‐type and mutant LIR and UBA domain constructs. (E) A2780 cells transfected with wt‐p62 and, LIR 338‐p62 or UBA 417‐p62 were treated with 4.5 µg/ml cisplatin for 12 hours and the expressions of Caspase 3 and Caspase 8 were analysed by western blotting. (F) A2780 cells were treated as E and Caspase 8 activity was analysed. Data are presented as mean ± SD, n = 3. *P < 0.05 vs wt‐p62

The UBA domain in p62 is a dimerization domain as well as ubiquitin‐binding domain and previous studies revealed that dimerization spatially occludes ubiquitin binding.^19^ Another report showed that expression of a p62 mutant deleted for the UBA domain reduced the level of apoptosis induced by HAMLET.^20^ To determine how p62 participates in Caspase 8 activation, we overexpressed either wild‐type p62 or the UBA truncated p62 mutant in SKOV3 and A2780 cells and then treated the cells with cisplatin. MTT assay showed that expression of the UBA truncated p62 mutant in cells treated with cisplatin resulted in significantly increased cell viability in both cell lines compared with expression of wild‐type p62 (Figure 3A,B). Moreover, Hoechst staining revealed that compared with wt‐p62, the nuclear fragmentation induced by cisplatin treatment was significantly reduced in cells overexpressing the UBA mutant compared with wild‐type p62 (Figure 3C,D). Annexin‐V FITC/PI staining, as well as evaluation of Caspase 3 activity, further validated that the UBA truncated p62 mutant that lacked the ability to bind ubiquitinated proteins reduced cisplatin‐induced apoptosis (Figure 3E‐G). Furthermore, we found that overexpressed wild‐type p62 in ovarian cancer cells treated with cisplatin significantly increased activation of Caspase 8 particularly in A2780 cells compared with overexpression of the UBA truncated p62 mutant (Figure 4A‐C).

During autophagy, cytoplasmic proteins are taken up into autophagic vesicles through autophagic receptors that contain a functionally conserved LIR domain. The LIR domain of p62 interacts with LC3, which is bound to the autophagic membrane, thus anchoring p62‐bound ubiquitinated proteins to the autophagosome.^21^ To further investigate the mechanism of p62 in Caspase 8 activation, we constructed two point mutants: one construct with a mutation in residue 338 in the p62 LIR domain, which abolishes the binding of p62 to LC3^23^ and another construct with a mutation in residue 417 in the UBA domain, which abolishes the binding of ubiquitinated proteins^24^ (Figure 4D). These point mutants have the advantage of minimizing the effect of domain changes on other functions of p62. We found that the Caspase 8 activation was significantly inhibited in A2780 cells transfected with the mutant p62 construct treated with cisplatin (Figure 4E,F), indicating that p62 binding to ubiquitinated proteins and to LC3 are required for Caspase 8 activation.

### 3.4 Autophagy degradation is involved in p62‐mediated activation of Caspase 8 in ovarian cancer cells

**Figure 5:**
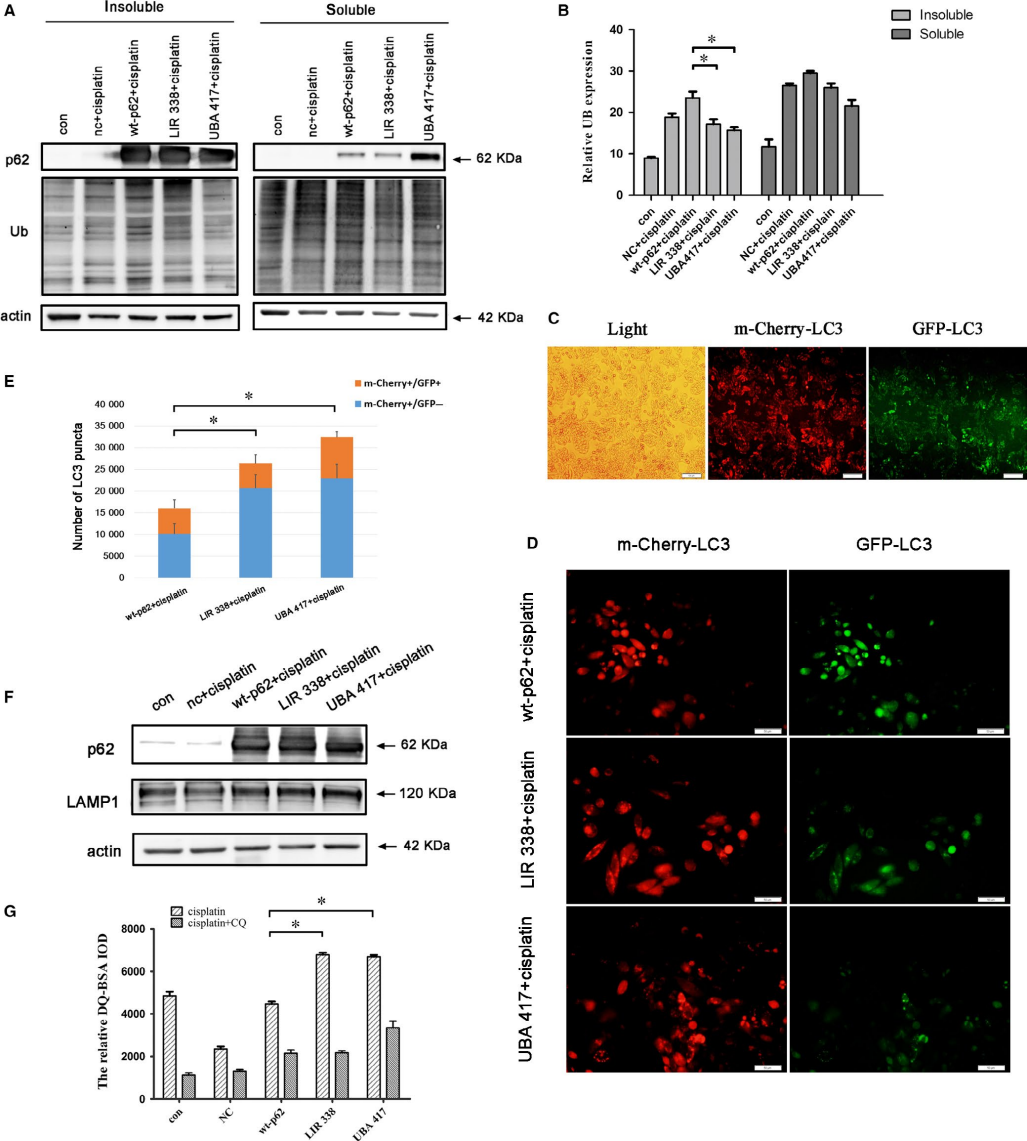
p62 regulates autophagic degradation in ovarian cancer cells. (A,B) SKOV3 cells transfected with wt‐p62, LIR 338‐p62 or UBA 417‐p62 were treated with 6 µg/ml cisplatin for 12 hours. Cells were lysed in 1% Triton X‐100 and Triton soluble and insoluble fractions were immunoblotted for anti‐ubiquitin, anti‐p62 and anti‐actin. (C‐E) SKOV3‐mCherry‐GFP‐LC3 cells were transfected with wt‐p62, LIR 338‐p62 or UBA 417‐p62. After treatment with 6 µg/ml cisplatin for 12 hours, fluorescence was visualized using microscopy. Scale bar, 50 µm. Data are presented as mean ± SD, n = 3. **P < 0.01. (F) After transfection with wt‐p62 or mutant p62, SKOV3 cells were treated with cisplatin (6 µg/mL) for 12 hours. Cell lysates were examined using immunoblotting with anti‐p62 and anti‐LAMP1. (G) SKOV3 cells were incubated with DQ‐BSA for 30 minutes at 37°C followed by a 2 hours chase in complete media. Lysosomal degradation as measured by DQ‐BSA dequenching was analysed by fluorescence microscopy. When indicated, chloroquine (25 µM) was used to block lysosome function. Data are presented as mean ± SD, n = 3. **P < 0.01

Previous studies reported that insoluble aggregated proteins are mainly degraded by autophagy.^25^ Other reports showed that the major components of insoluble fractions are aggresomes with ubiquitin conjugates that are substrates for autophagic degradation.^26^ We found that the overexpression of wild‐type p62 led to an increased amount of insoluble ubiquitinated protein accumulation, which was similar to the accumulation observed with autophagy inhibition caused by chloroquine treatment (Figure 2K), In contrast, the main part of ubiquitinated proteins were in the soluble fractions with p62 mutants transfection (Figure 5A,B).

Our findings indicated that p62 promotes Caspase 8 activation and requires domains that are closely associated with autophagic degradation. To examine the impact on autophagic flux, we used the tandem mCherry‐GFP‐LC3 reporter assay. GFP is quenched rapidly in acid lysosomes, but mCherry is not; thus mCherry‐only puncta correspond with mature autolysosomes, whereas double‐positive mCherry/GFP puncta correspond with early autosomes.^27^ Our results revealed that compared with wild‐type p62, there were higher numbers of mature autophagosomes in SKOV3 cells transfected with the p62 mutants treated with cisplatin for 12 hours and 24 hours (Figure 5C‐E, Figure S1). As completion of autophagy involves fusing with lysosomes, we examined lysosome degradation using DQ Red BSA, a fluorescent probe that tracks through the endosomal pathway and is ultimately de‐quenched following proteolytic cleavage in the lysosome.^2^ We found that expression of p62 mutants lacking the LIR and UBA domains resulted in increased lysosome degradation and this degradation was suppressed by chloroquine treatment (Figure 5F). However, no obvious changes in the levels of the lysosomal protein Lamp1 were observed among the transfection groups, which suggested that the number of lysosomes may not be increased (Figure 5G). These results suggest that p62 LIR and UBA functional domains are involved in the regulation of autophagic degradation.

### 3.5 p62 functions as a bridge for the recruitment and activation of Caspase 8 on autophagosome membranes in ovarian cancer cells

**Figure 6:**
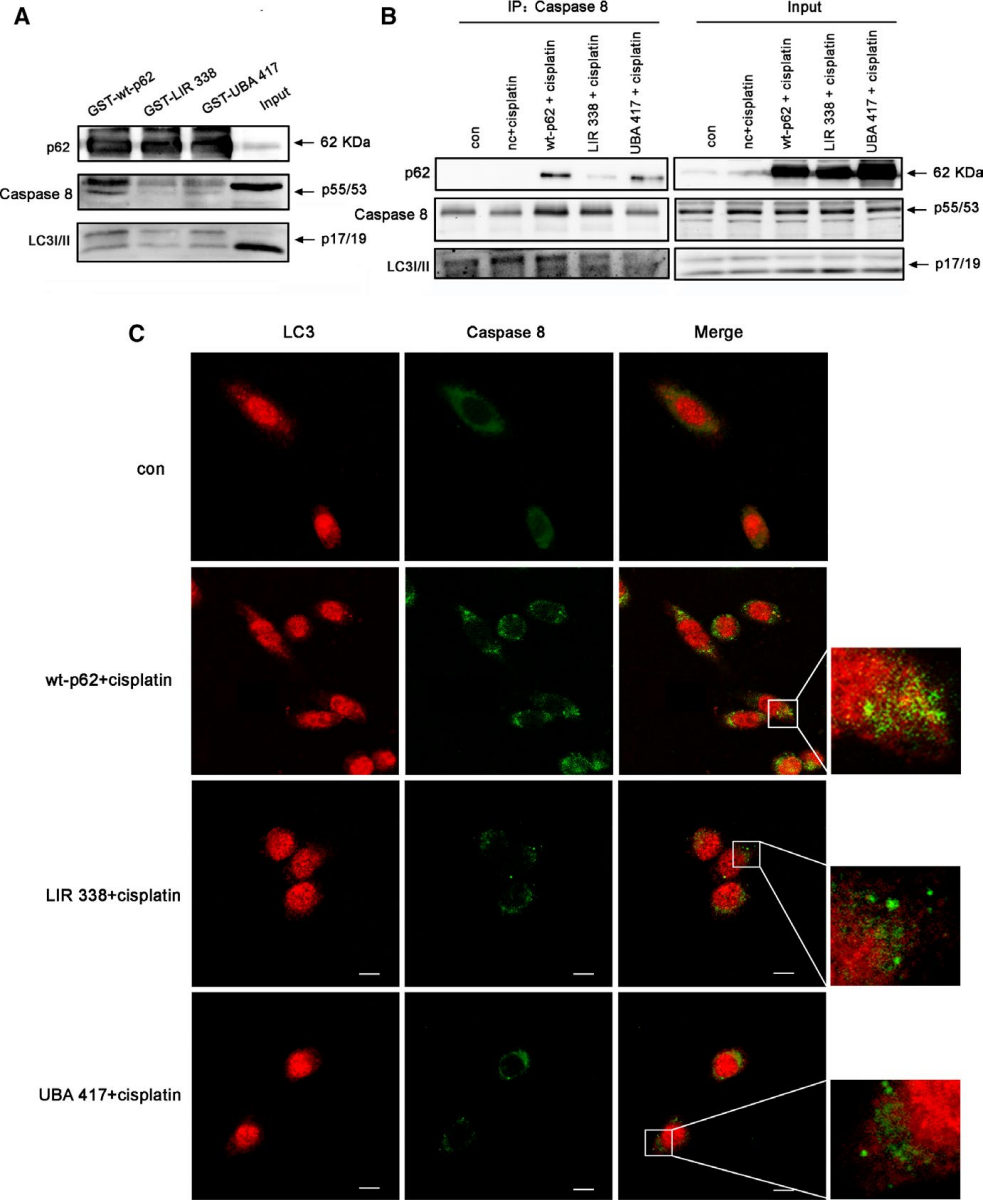
p62 promotes co‐localization of Caspase 8 with the autophagy membrane and mediates Caspase 8 activation in cisplatin‐treated ovarian cancer cells. (A) GST‐p62 fusion proteins purified using GST affinity chromatography were used in pull‐down experiments with lysates from tumours from the A2780 cell nude mouse xenograft model. Immunoblotting was performed with anti‐p62, anti‐Caspase 8 and anti‐LC3. (B) SKOV3 cells transfected with wt‐p62 or mutant p62 were treated with cisplatin (6 µg/mL) for 12 h. Immunoprecipitation was performed with the anti‐Caspase 8 antibody followed by western blotting using anti‐p62, anti‐Caspase 8, anti‐LC3 and anti‐ubiquitin antibody. (C) SKOV3 cells were treated as in B. Localization of Caspase 8 and LC3 was observed with confocal laser microscopy. Scale bar, 10 µm

Yong et al showed that the cleavage and activation of Caspase 8 requires the autophagosomal membrane.^29^ To further dissect the role of p62 and autophagy in the activation of Caspase 8, we performed GST pull‐down assays and detected the binding of GST‐wild‐type‐p62 and GST‐p62 protein domain mutants to Caspase 8 from cisplatin‐treated tumour tissues from xenografts. We found that wild‐type p62 specifically interacted with Caspase 8 and LC3, whereas the p62 LIR and UBA domain mutants did not (Figure 6A). Co‐immunoprecipitation and immunofluorescence confirmed that Caspase 8 and LC3 showed increased co‐localization in SKOV3 cells transfected with wild‐type p62 compared with cells expressing mutant p62 (Figure 6B,C). These results indicated that p62 functions as a bridge for the recruitment of Caspase 8 on the autophagosome membrane in ovarian cancer cells treated with cisplatin and this recruitment and activation of Caspase 8 was inhibited by mutation of key domains in p62.

## 4 DISCUSSION

**Figure 7:**
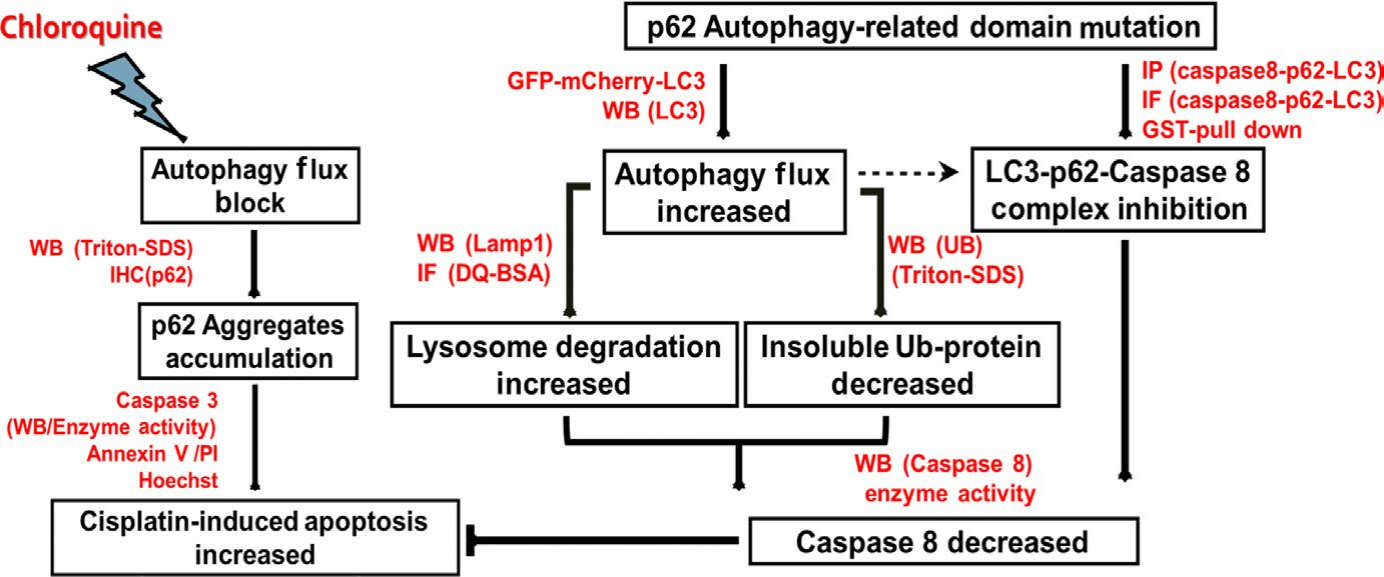
Proposed model by which the accumulated p62 promotes Caspase 8 activation during cisplatin‐induced apoptosis when autophagic flux is suppressed

In this study, our data indicated that high expression of p62 and Caspase 8 were associated with longer survival time and less relapse in ovarian cancer. The insoluble p62 and ubiquitinated protein aggregates caused by autophagic flux suppression at the late stage of autophagy increased the activation of Caspase 8. This ‘ubiquitin stress’ promoted the recruitment of pro‐Caspase 8 by p62 functional domain UBA and LIR and amplified the apoptotic signal, which promoted the cisplatin‐induced death through a non‐canonical Caspase 8 activation pathway (Figure 7).

Recent evidence has confirmed a critical role for p62 in ovarian cancer progression. Iwadate assessed p62 expression in 266 primary epithelial ovarian cancer tissues and showed that high expression of p62 was correlated with poor prognosis. However, another study in 2018 suggested that p62 overexpression indicates favourable prognosis.[1,2] Our study attempted to reconcile these observations by clarifying a potential interacting functional partner of p62. Caspase 8‐mediated apoptosis signalling activation is dependent on its cleavage and is regulated by ubiquitination.^30^ Analysis of death‐associated proteins by MS/MS revealed that p62 can bind Caspase 8.^12^, ^31^ Huang et al confirmed that up‐regulation of p62 in colorectal cancer significantly increased Caspase 8 accumulation by BCL‐2 inhibitors.^32^ Our data showed that high expression of both p62 and Caspase 8 was associated with longer survival time and less relapse in ovarian cancer. To further determine the role of aggregated p62 in Caspase 8 activation, we examined the effect of the combination of cisplatin and chloroquine which increased p62 and ubiquitinated protein accumulation. These results showed that Caspase 8 was significantly up‐regulated and tumour cell growth was inhibited in vivo and in vitro, which indicated that p62 may act as a tumour suppressor through recruitment and activation of Caspase 8.

As a substrate for ubiquitinated protein and a substrate for selective autophagy, p62 recognizes ubiquitinated proteins and brings them to the autophagic membrane through its UBA domain, whereas p62 binds LC3 through its LIR domain.^33^, ^34^ Our results showed that the UBA truncated p62 mutant could inhibit the activation of Caspase 8 in response to cisplatin in ovarian cancer cells. We confirmed similar results using the p62 UBA 417 point mutant. These results suggested that p62 regulation of Caspase 8 activation was related to its ability to bind ubiquitinated proteins. Recently, investigators found that autophagosome accumulation caused by Atg2A/B deficiency promoted the activation of Caspase 8 in response to nutrient starvation in THP‐1 and Hela cells, which indicated that autophagy was involved in cell death caused by Caspase 8 activation.^35^ Consistent with these findings, our data suggested that Caspase 8 activation in response to cisplatin was also suppressed by the p62 mutant that was unable to bind LC3. We used co‐immunoprecipitation and immunofluorescence experiments to examine the localization of Caspase 8 and LC3 and found that the UBA and LIR domain p62 mutants showed weakened interaction with the autophagic membrane. We confirmed this finding in tumour tissues from xenografts through GST pull‐down assays. Together these findings suggested that p62 mediates Caspase 8 activation in response to cisplatin in an ubiquitin‐binding and autophagic membrane‐dependent manner in ovarian cancer cells.

Autophagic flux is a process that starts with the stepwise engulfment of cytoplasmic material targeted for degradation by the isolation membrane.^36^ Distinguished from ‘autophagic death’ was reported to take place under conditions of defective apoptotic machinery, the initiation of cell death is triggered by the blocking of protective autophagy in this study.^37^ We showed that p62 accumulation induced by autophagy inhibition can increase the sensitivity of ovarian cancer cells to cisplatin in vitro and in vivo. Although we demonstrated the role of high expression of p62 and Caspase 8 in ovarian cancer, there are still many issues to be further investigated. For example, although p62 may recruit various partners under different pathological conditions the regulatory mechanisms underlying the recruitment and binding steps are still unknown. Furthermore, these results suggest that activation of Caspase 8 is regulated by ubiquitin, but the role of deubiquitinating enzymes in p62‐mediated Caspase 8 activation has not been determined. Further studies should address these questions to fully clarify the function of p62 in ovarian cancer.

In summary, our study has characterized the role of p62 in ovarian cancer. We found that high expression of p62 and Caspase 8 is associated with favourable prognosis and progression of ovarian cancer. p62 promotes the recruitment and activation of Caspase 8 on autophagic membranes through the autophagy‐related domains in p62, which establish a crosstalk between autophagy and apoptosis through p62 and Caspase 8. p62/Caspase 8 may become promising prognostic biomarkers and onctargets for ovarian cancer treatment.

## CONFLICT OF INTEREST

The authors declare that they have no potential conflicts of interest.
