# Supplementary figures and images for: p62 aggregates mediated Caspase 8 activation is responsible for progression of ovarian cancer
Source: J Cell Mol Med. 2019 Apr 2;23(6):4030–42. doi: 10.1111/jcmm.14288 (PMC6533521; doi:10.1111/jcmm.14288)

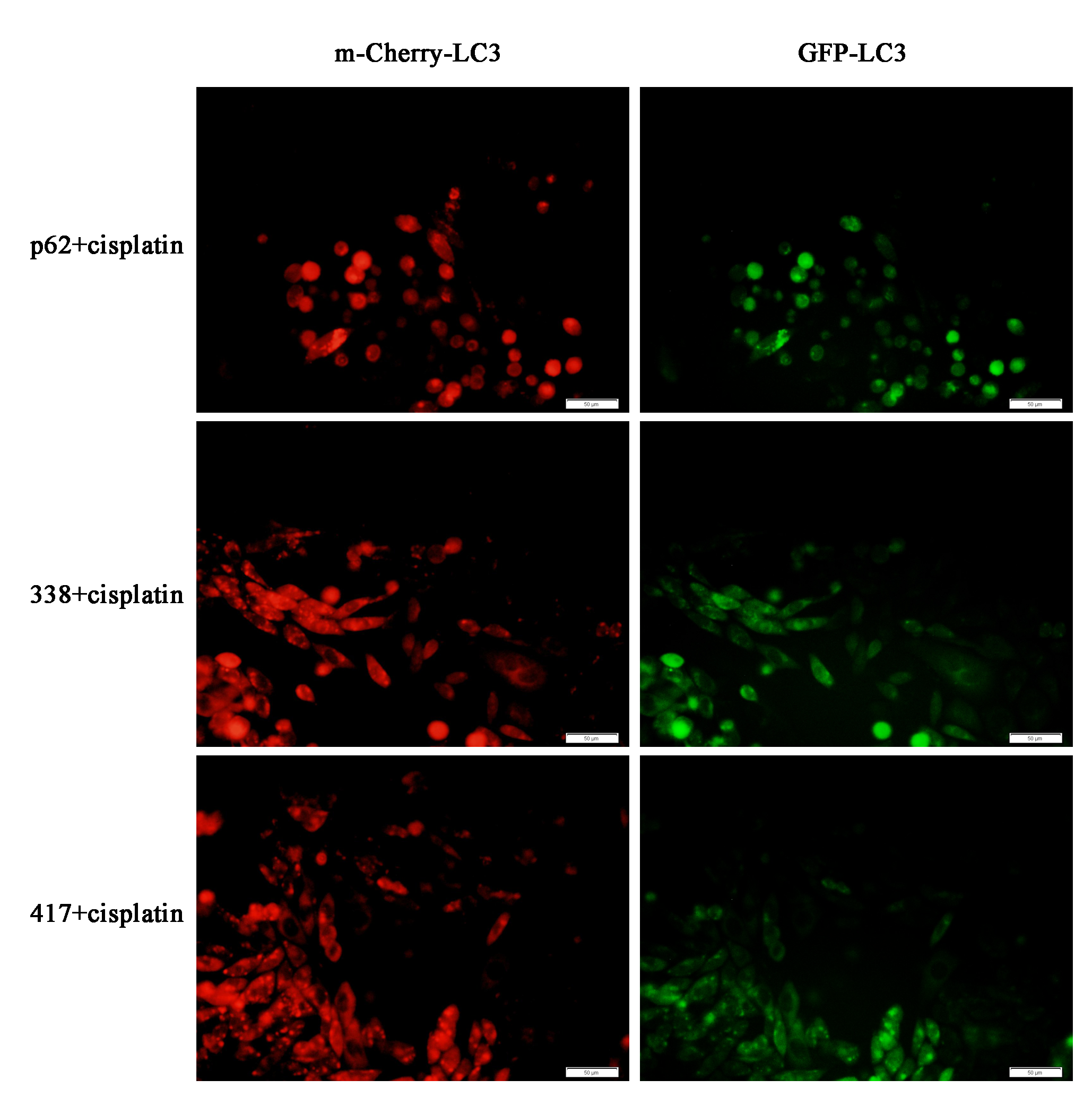

Supplement: Supplementary file 1 [file JCMM-23-4030-s001.tif]
